# Heterogeneity between insulin and proinsulin in the potency for insulin autoantibodies in newly diagnosed type 1 diabetes children

**DOI:** 10.1093/cei/uxag026

**Published:** 2026-05-05

**Authors:** Pantea Parsian, Rasmus Bennet, Cheng-ting Tsai, Anita Ramelius, Åke Lernmark, Josefine Jönsson, the BDD Study Group, Alexander Lind, Samia Hamdan, Annelie Carlsson, Helena Elding Larsson, Auste Lyckå, Gun Forsander, Martina Persson, Johnny Ludvigsson, Karin Åkesson

**Affiliations:** Department of Clinical Sciences, Lund University CRC, Skåne University Hospital, Malmö, Sweden; Department of Clinical Sciences, Lund University CRC, Skåne University Hospital, Malmö, Sweden; The Research and Development Laboratory, Enable Biosciences, Inc., South SanFrancisco, CA, USA; Department of Clinical Sciences, Lund University CRC, Skåne University Hospital, Malmö, Sweden; Department of Clinical Sciences, Lund University CRC, Skåne University Hospital, Malmö, Sweden; Department of Clinical Sciences, Lund University CRC, Skåne University Hospital, Malmö, Sweden

**Keywords:** insulin, proinsulin, autoantibodies, antibody dete ction by agglutination PCR, IC50, potency

## Abstract

Autoantibodies against insulin (IAA) are early appearing markers of autoimmunity against the pancreatic islet beta cells and predict progression to type 1 diabetes if additional islet autoantibodies also develop. It is still controversial if proinsulin rather than insulin is the primary autoantibody. The aim of the present study was to compare the half-maximal concentration (IC50) between insulin and proinsulin to displace the binding of insulin to insulin autoantibodies (IAA) in the Antibody Detection by Agglutination PCR (ADAP) assay. IC50 as a measure of potency to displace insulin binding to IAA was determined in 36 newly diagnosed type 1 diabetes children. The ability of either insulin or proinsulin to displace IAA was heterogenous. Proinsulin curves were consistently right-shifted relative to insulin as median IC50 was 21.0 nM (IQR 14.9–25.1) for insulin and 26.1 nM (IQR 12.3–37.5) for proinsulin. A significant age × sex interaction was observed (F (1,31) = 14.3, *P* < 0.001), indicating that IC50 for both insulin and proinsulin increased with age in boys but decreased in girls. It may reflect whether autoantibodies to insulin or proinsulin were first appearing or of variable maturation from the time of initiation through progression to clinical onset. It was concluded that the IC50 of insulin and proinsulin for IAA was comparable in children with newly diagnosed type 1 diabetes. The ADAP IAA assay should prove useful to determine whether insulin or proinsulin is the primary target at the time of seroconversion to IAA.

## Graphical Abstract

**Graphical Abstract:**
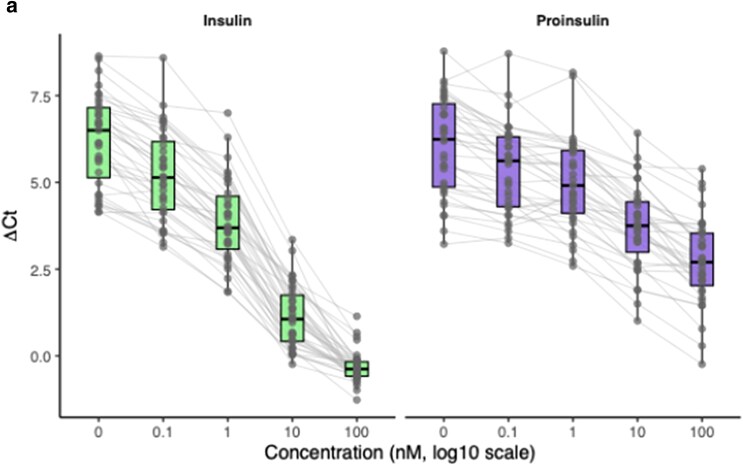

## Introduction

Autoimmune (type 1) diabetes (T1D) developed after the pancreatic islet beta cells are destroyed in prolonged autoimmunity resulting in the loss of insulin production [1]. In children with increased genetic risk for T1D who were followed from birth, it was revealed that the first appearing autoantibody was to insulin (IAA) in 1–4 year olds, while the first appearing autoantibody to glutamic acid decarboxylase (GADA) appeared later after 2–3 years of age, thereby representing two different phenotypes [2, 3]. Interestingly, in the TEDDY study, which followed children from birth to 15 years of age, 39% had IAA-first, 41% had GADA-first, and 13% had both at the same time, while the remaining 7% had various combinations including autoantibodies to IA-2 (IA-2A) [4]. Children who retain only one autoantibody have about 15% risk for diabetes within 10 years, while the risk for children with two or more autoantibodies amounts to 85% [5, 6].

The European Medicines Agency (EMA) has approved islet autoantibodies as biomarkers for T1D in prevention clinical trials [7]. The incidence rate of IAA peaked already at 1–2 years of age [2,3] . A second autoantibody, mostly GADA, developed within 1 year in about 60% of the IAA-first children [6]. However, in individuals at risk for T1D, a proliferative T-cell response to proinsulin was observed more frequently than a response to insulin [8]. Using insulin and proinsulin, respectively, labelled with ^125^I in radiobinding assays, proinsulin autoantibodies (PIAA) were more common than IAA in newly diagnosed T1D patients [9]. However, others found that IAA was more specific than PIAA for prediction of T1D [10]. In children born to parents with T1D, all high-affinity IAA detected prior to T1D diagnosis were reactive with proinsulin, while most lower-affinity IAA did not bind proinsulin [11]. In children with multiple islet autoantibodies who progressed to T1D had higher affinity IAA than children who did not develop multiple islet autoantibodies or progress to T1D [12]. It therefore remains unclear whether the autoantibody response is primarily triggered against insulin, proinsulin, or both.

IAA is detected in a radiobinding assay with A^14^-labeled ^125^I-insulin using Sepharose-Protein A/G to separate antibody-bound from free labeled insulin [13]. The IAA assay is routinely subjected to international workshop standardization [14, 15]. However, the performance of the IAA radiobinding assay remains heterogeneous and consistently shows high variability across laboratories. Among novel nonradioactive IAA immunoassays, the antibody detection by agglutination-PCR (ADAP) [16, 17] assay showed a consistently better performance over the IAA radiobinding assay [15, 18]. The ADAP-IAA assay was validated in children with newly diagnosed T1D in the Swedish Better Diabetes Diagnosis (BDD) study and healthy controls to demonstrate improved diagnostic sensitivity and specificity for IAA [19]. In this study, a competitive displacement analysis of ADAP-IAA was done with cold insulin and proinsulin, respectively, in randomly selected serum samples positive for IAA [19]. The study design was to determine the ability of insulin and proinsulin to displace binding to IAA present at the time of clinical diagnosis of T1D. IC50 (half-maximal inhibitory concentration) was used to measure the potency of either insulin or proinsulin in inhibiting the binding of the labeled to IAA by 50%.

The aim was to test the hypothesis that diagnostic sensitivity would be higher for IAA with potency for insulin rather than proinsulin. A lower IC50 means higher potency as less cold ligand (insulin or proinsulin) is needed to displace IAA binding.

## Materials and methods

### Research participants

The serum samples used in the study were from the BDD study of children aged 1–18 years of age who were newly diagnosed with T1D [20]. The samples were obtained 3–5 days after the initiation of insulin therapy. Serum samples from a total of 36 patients positive for IAA in the ADAP assay [19] were selected to be displaced by cold insulin or proinsulin. Age, sex, HLA-DQ haplotypes, ADAP-IAA levels as well as ADAP detected autoantibodies against glutamic acid decarboxylase (GADA), IA-2 (IA-2A) or ZnT8 transporter (ZnT8A) are summarized in Table 1. Besides these samples, quality control samples (high, medium, low, and negative) were routinely included in each run of the ADAP assay [19].

**Table 1:** Insulin autoantibodies (IAA) and other islet autoantibodies determined by ADAP in newly diagnosed type 1 diabetes children.

Sample number	Age (years)	Sex	HLA-DQ haplotype-1	HLA-DQ haplotype-2	IAA* ΔCt	IAA	GADA	IA-2A	ZnT8A
01	3.66	F	A1*05-B1*02	B1*03:02	7.00	Pos	Pos	Pos	Pos
02	5.66	M	A1*05-B1*02	B1*03:02	7.11	Pos	Pos	Pos	Neg
03	1.56	F	B1*03:02	B1*06:04	8.19	Pos	Pos	Pos	Neg
04	1.03	F	A1*05-B1*02	B1*03:02	8.02	Pos	Pos	Pos	Neg
05	2.82	M	B1*03:02	B1*03:02	7.90	Pos	Pos	Pos	Pos
06	2.22	F	A1*05-B1*02	B1*03:02	9.09	Pos	Pos	Pos	Pos
07	5.80	F	B1*03:02	B1*03:02	7.44	Pos	Pos	Pos	Neg
08	7.89	F	B1*03:02	B1*05:01	8.01	Pos	Pos	Neg	Neg
09	4.54	M	A1*05-B1*02	B1*03:02	6.88	Pos	Pos	Pos	Pos
10	2.53	M	B1*03:02	B1*05:01	7.97	Pos	Pos	Pos	Neg
11	2.63	F	A1*02-B1*02	B1*06:04	7.65	Pos	Pos	Pos	Pos
12	9.14	F	B1*03:02	B1*03:02	7.81	Pos	Pos	Neg	Neg
13	1.28	F	A1*05-B1*02	B1*03:03	7.23	Pos	Pos	Neg	Neg
14	8.86	F	B1*03:02	B1*03:02	6.90	Pos	Pos	Pos	Neg
15	8.04	F	A1*05-B1*02	A1*02-B1*02	7.43	Pos	Pos	Pos	Pos
16	5.71	M	B1*03:02	B1*06:03	7.70	Pos	Pos	Neg	Neg
17	8.15	M	A1*05-B1*02	A1*02:01-B1*02	6.84	Pos	Pos	Pos	Neg
18	6.39	M	A1*05-B1*02	B1*03:02	7.58	Pos	Pos	Pos	Pos
19	2.28	F	B1*03:02	B1*05:01	8.45	Pos	Pos	Pos	Neg
20	3.45	M	B1*03:02	B1*03:02	7.01	Pos	Pos	Pos	Pos
21	9.74	F	B1*03:02	B1*04	6.76	Pos	Pos	Pos	Neg
22	3.27	M	A1*05-B1*02	B1*03:02	8.35	Pos	Pos	Pos	Pos
23	7.48	M	A1*05-B1*02	B1*03:01	7.07	Pos	Pos	Pos	Neg
24	2.21	F	A1*05-B1*02	B1*03:02	8.11	Pos	Pos	Pos	Neg
25	8.10	M	A1*02-B1*02	B1*03:02	7.58	Pos	Pos	Pos	Pos
26	1.46	F	B1*03:02	B1*05:01	7.80	Pos	Pos	Pos	Pos
27	7.56	M	A1*05-B1*02	B1*03:01	7.82	Pos	Pos	Pos	Neg
28	4.28	M	A1*05-B1*02	B1*03:02	7.23	Pos	Pos	Pos	Pos
29	1.61	M	A1*05-B1*02	B1*03:02	7.44	Pos	Pos	Pos	Pos
30	1.54	F	B1*03:03	B1*04	7.16	Pos	Pos	Pos	Neg
31	2.69	M	A1*05-B1*02	B1*03:02	6.92	Pos	Pos	Pos	Neg
32	4.63	F	A1*05-B1*02	B1*03:02	7.42	Pos	Pos	Pos	Pos
33	1.94	M	A1*05-B1*02	B1*03:02	7.54	Pos	Pos	Pos	Pos
34	2.98	F	N/A	N/A	9.63	Pos	Pos	Neg	Neg
35	1.47	F	B1*03:02	B1*06:04	8.04	Pos	Pos	Pos	No data
36	10.44	F	A1*05-B1*02	B1*03:02	7.07	Pos	Pos	Pos	No data

^*^ΔCt data used to select available ADAP IAA positive samples in the Better Diabetes Diagnosis study [19].

Summary for the text:

DQ2/8 15

DQ2 but not 8 5

DQ8 but not 2 13

Neither 2, nor 8. 1

N/A is not analyzed

### ADAP assay reagents

The synthesis oligonucleotide-conjugated insulin providing a tracer concentration of 0.5 nM was described previously [18]. All reagents for the ADAP assay, except for the molecular grade H_2_O, were organized in kits for single plex IAA and were produced by Enable Biosciences, Inc. (South San Francisco, CA, USA).

### Dilution of patient serum samples

Each serum sample (60 µl) was serially diluted in 60 µl buffer C (PBS (pH 7.4) with 8 mmol/L EDTA, 0.2% (v/v) Triton X-100 and 2% (w/v) BSA) with insulin (Actrapid®, Novo Nordisk A/S, Copenhagen, Denmark containing 3.47 mg/ml recombinant human insulin) or recombinant human proinsulin (Catalog # 1336-PN, R&D Systems, Inc., Minneapolis, MN, USA) serially diluted to final concentrations of 0.086, 0.86, 8.6, and 86 nM for insulin and 0.053, 0.53, 5.3, and 53 nM for proinsulin. The samples were mixed with insulin or proinsulin, separately and frozen at −20 C until use within a day or 2.

### ADAP assay of diluted patient serum samples

A customized Hamilton Microlab STAR (Hamilton, Bonaduz, Switzerland) was used for the ADAP assay [21] allowing a maximum of 80 samples to be analyzed in each run [18, 19]. Sera from eight different patients was analyzed per run. Insulin and proinsulin displacement was carried out on the same plates in duplicate determinations. In each ADAP assay, a conjugation mix, ligation mix, pre-amplification mix, and qPCR mix were made. The principle of the ADAP assay is that antigen-specific DNA conjugates bind to the corresponding antibodies in the serum sample, which enables the cascade of agglutination, ligation, pre-amplification, and quantification by PCR, leading to a positive test result [16]. In this competitive displacement study, the added insulin or proinsulin in the serum would compete with the tracer DNA conjugate in binding to the IAA.

#### Conjugation mix

In a 1.7 ml microcentrifuge tube, 427.6 µl buffer C was added as well as 214 µl buffer A2, 171 µl ssDNA, 85 µl IgG, 35.6 µl conjugate Ins-3A, and 35.6 µl conjugate Ins-3B. The tube was vortexed, spun down on the tabletop microcentrifuge, labeled, and stored on ice.

#### Ligation mix

DTT (three tubes of dithiothreitol dry powder) was dissolved each in 200 µl molecular grade H_2_O and was left to sit at room temperature for 10–15 minutes to dissolve. The tubes were vortexed, combined, and then vortexed again. A total of 9667 µl molecular grade H_2_O, 528 µl DTT solution, 628 µl bridge oligo, 1255 µl 10 × ligation buffer, and 63 µl ampligase were added to a 15 ml conical tube, which was inverted and stored on ice before use.

#### Pre-amplification mix

The mixture was made by adding 1196 µl molecular grade H_2_O, 1285 µl 5xHS PCR buffer, 193 µl MgCl_2_, 512 µl dNTP, and 39 µl 2 µM primer (3 FR) to a 5 ml conical tube, which was vortexed and microcentrifuged. The mixture was divided into two 1.7 ml centrifuge tubes and stored on ice.

#### qPCR-primer mix

The reagents for the qPCR-master mix were kept dark, and 96 µl 10 µM primer, 96 µl molecular grade H_2_O, and 1200 µl qPCR master mix were added to a 1.7 ml microcentrifuge tube, which was inverted five times to mix and then centrifuged.

Each of the four control samples (20 µl) was added to 1.7 ml microcentrifuge tubes. Buffer C (100 µl) was added to a 1.7 ml microcentrifuge tube as a blank to determine the limit of detection of the ADAP assay [15]. The ligation mix was poured in the first position of the sample reservoir, and 14–15 ml molecular grade H_2_O was poured in the fifth position of the reservoir.

### Calculation and reporting of assay results

The antibody levels in the ADAP assay were expressed as delta Ct (ΔCt) i.e. the number of PCR cycles [15], and the cutoff for a positive sample was 0.87 ΔCt. The serum dilution, which consisted of only serum and Buffer C, served as the reference point to be compared with the retained signals from the displacements with insulin and proinsulin, respectively. Maximal binding (100%) was at no insulin or proinsulin (0 nM) added.

IC50 was used to measure the potency of insulin compared to proinsulin in inhibiting IAA. It quantifies the concentration (nM) necessary to reduce binding by half its maximum value as a direct measure of efficacy. IC50 values were estimated from Kd using the Cheng–Prusoff equation (below), assuming equivalent binding affinity between native insulin and the insulin–DNA conjugate (Kd^L^ ≈ Kd) [22].


$$IC50=\;K_{d}\left(1+\frac{\left[L\right]}{K_{d}^{L}}\right)$$

### Data analysis

#### Quality control and dataset

Quality control procedures were applied to all displacement experiments. Of the 36 serum samples analyzed for proinsulin-mediated displacement, eight samples (01, 03, 05, 07, 08, 21, 31, and 33) were reanalyzed because five initially showed unusually high proinsulin IC50 values, and three showed no measurable displacement at the tested concentrations. Upon repeat analysis, seven samples yielded evaluable displacement curves, and these repeat measurements were used in all subsequent analyses and figures. Sample 08 again showed no measurable displacement by proinsulin and was excluded from all analyses and figures. The final analytical dataset thus comprised 35 participants.

#### IC50 calculation and transformation

All IC50 values were log10-transformed prior to inferential analyses to account for skewness and to allow interpretation on a relative (fold-change) scale. IC50 values are reported in nM; log10-transformed IC50 values are used for statistical analyses. Within-individual differences between proinsulin and insulin IC50 values were calculated as


$$\Delta log_{10}(IC50)=\;log_{10}(IC50_{\;proinsulin})-\;log_{10}(IC50_{insulin})$$

Paired differences were assessed using the Wilcoxon signed-rank test.

#### Associations with age and sex

Associations between IC50 and age at diagnosis, sex, and ligand type (insulin or proinsulin) were evaluated using linear mixed-effects models with log10(IC50) as the outcome. Sample ID was included as a random intercept to account for repeated measurements (insulin and proinsulin per individual). Fixed effects included age (mean-centered), sex, ligand type, and relevant interactions. Statistical significance of fixed effects was determined using type III ANOVA with Satterthwaite’s approximation for degrees of freedom.

#### Associations with HLA and autoantibody status

Associations between IC50 and HLA genotype or autoantibody status were assessed using separate linear regression models with log10(IC50) as the outcome. Sensitivity analyses included (i) exclusion of individuals with incomplete autoantibody data and (ii) collapsing autoantibody groups. Sex was considered as a covariate but did not materially change results and was not included in the final models. Differences in Δlog10(IC50) across sex were evaluated using the Wilcoxon rank-sum test, and across HLA or autoantibody groups using the Kruskal–Wallis test.

#### Relationship with ADAP IAA levels

Associations between ADAP IAA levels (ΔCt) and IC50 were assessed using linear mixed-effects regression models with log10(IC50) as the outcome and ADAP ΔCt as the predictor. Sample ID was included as a random intercept to account for repeated measures. Sex was considered as a covariate but did not materially change results and was not included in the final models.

#### Data visualization

Displacement curves were plotted on a logarithmic concentration scale (nM) with normalized binding on the *y*-axis. Group comparisons were visualized using boxplots with overlaid individual points; paired comparisons were also displayed within-individual connections. Associations between continuous variables were illustrated with scatterplots and fitted regression lines as appropriate. All statistical analyses were performed in R (version 4.4.3), and all figures were generated using ggplot2. A two-sided *P*-value < 0.05 was considered statistically significant.

## Results

The displacement curves were comparable for all samples except for sample number 23, where ΔCt was slightly higher at 0.5 ng/ml insulin (4.77) compared to ΔCt at 0 ng/ml insulin (4.63) (Table 2).

**Table 2:** ADAP IAA (Δct) with and without displacement with 0–86 nM human recombinant insulin.

Sample number	Insulin displacement concentration (nM)				
	0	0.086	0.86	8.6	86
01	4.98	4.26	2.83	1.11	−0.08
02	6.13	5.17	4.32	1.06	−0.29
03	7.12	6.55	4.68	2.29	−0.13
04	7.55	6.30	4.37	1.21	0.02
05	6.72	5.43	3.63	0.96	−0.17
06	7.80	6.28	5.72	2.02	0.46
07	4.32	3.55	2.60	0.24	−0.26
08	5.85	4.57	2.30	−0.49	−0.72
09	4.17	3.14	1.86	0.02	−0.43
10	6.14	3.27	1.84	0.07	−0.59
11	8.22	6.77	4.98	1.34	−0.62
12	5.76	4.17	2.93	0.05	−0.17
13	5.59	4.92	3.24	1.36	−0.85
14	5.29	4.95	3.54	0.40	−0.38
15	6.77	5.89	3.99	2.16	1.14
16	7.19	6.70	5.10	1.79	−0.64
17	4.34	3.89	2.23	0.17	−1.27
18	5.67	4.55	3.74	0.24	−0.36
19	6.95	5.52	4.10	2.18	−0.36
20	4.65	3.60	2.51	−0.24	−0.76
21	4.14	4.12	2.76	0.44	−0.50
22	6.93	6.07	5.11	1.40	−0.55
23	4.63	4.77	3.59	0.63	−0.17
24	7.55	6.88	5.30	1.59	−0.19
25	5.67	5.14	3.31	0.59	−0.54
26	6.05	4.46	4.06	0.64	−0.61
27	7.41	6.78	5.23	1.71	−0.71
28	7.04	5.10	4.52	1.56	−0.48
29	6.65	4.59	3.30	0.68	−0.52
30	4.43	3.53	3.22	1.94	0.67
31	4.53	3.61	2.82	0.46	−0.32
32	7.31	5.68	4.11	1.05	−0.39
33	6.50	5.66	3.69	2.33	−0.11
34	8.56	7.22	7.00	3.35	0.56
35	8.64	8.59	6.30	3.03	−0.99
36	6.69	5.44	3.57	0.05	−0.58

### Displacement and IC50 values

All 36 ADAP-IAA samples (Table 1) were displaced by insulin. Thirty-five samples showed measurable displacement by proinsulin, whereas one sample (08) did not, suggesting possible insulin-specific reactivity. After repeat testing (see Methods), this sample remained nondisplaceable by proinsulin and was excluded. All subsequent analyses were performed in 35 participants.

**Figure 1:**
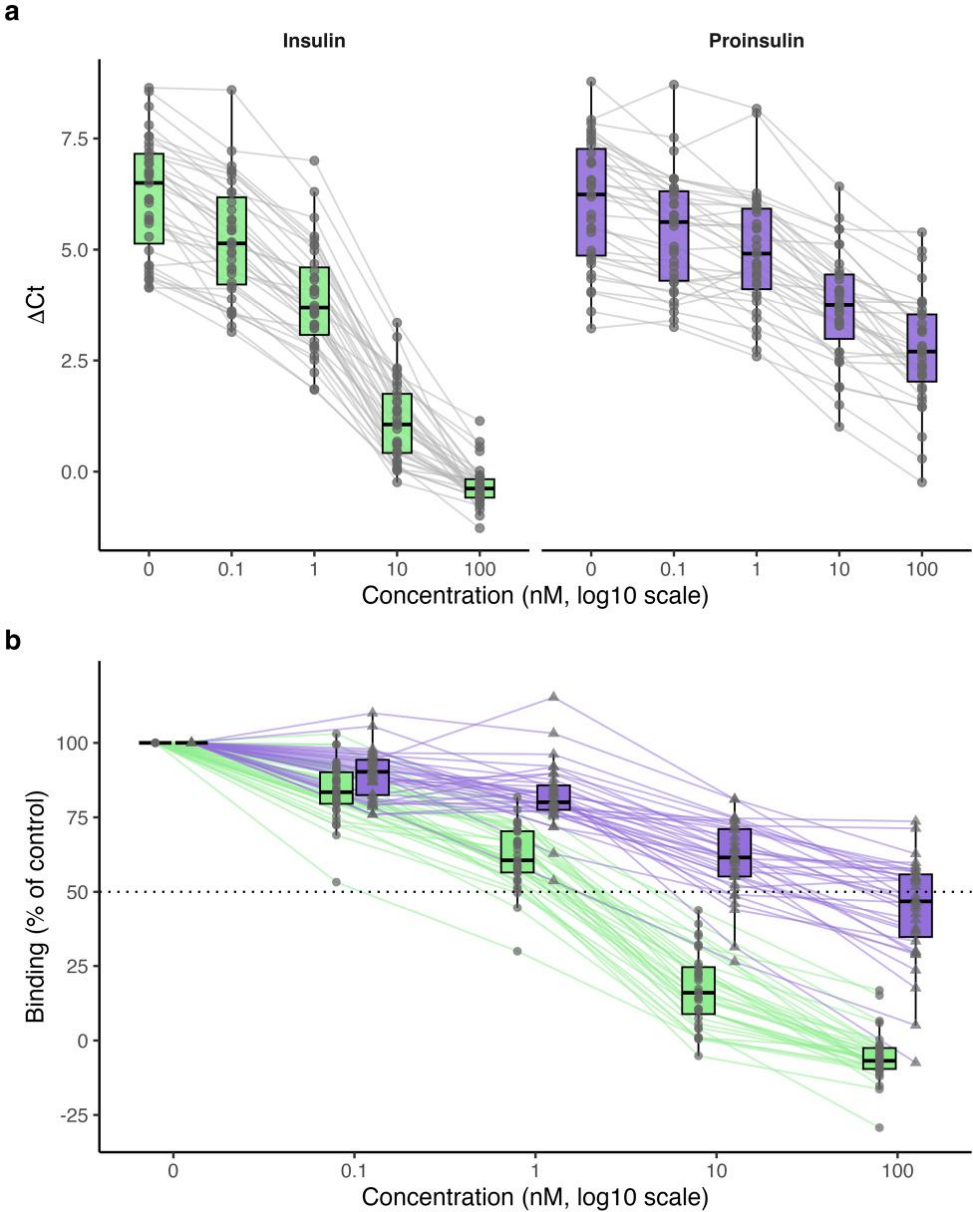
**Displacement of insulin autoantibody (IAA) binding by insulin and proinsulin in the ADAP assay. (**a**)** Displacement of IAA binding by increasing log10 concentrations (0–100 nM) of unlabeled insulin (green; actual concentrations 0–86 nM) or proinsulin (purple; actual concentrations 0–53 nM). ΔCt values are shown for each individual sample (grey lines), with boxplots summarizing the distribution at each concentration. Because log10(0) is undefined, the 0 nM condition is plotted at a nominal concentration of 0.01 nM for visualization on the logarithmic *x*-axis; all other concentrations are plotted at their true values. (b) The same displacement data normalized to percent binding, where 100% represents binding in the absence of competitor (0 nM). Each point represents an individual sample, with paired measurements connected by lines. The dotted horizontal line indicates 50% binding (IC50 reference level).

**Figure 2:**
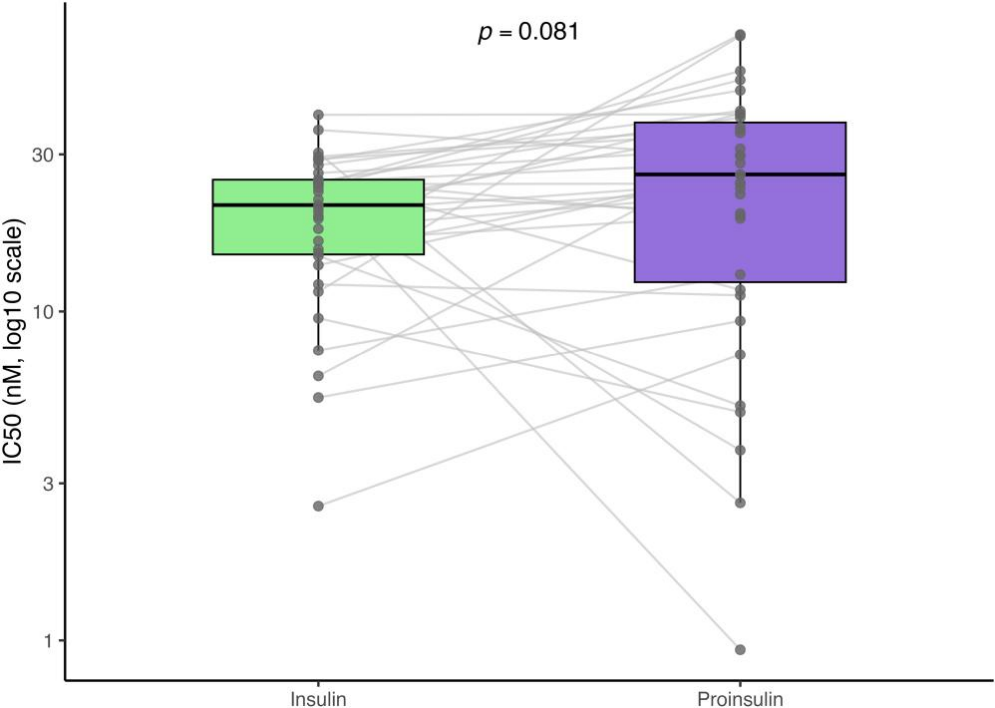
**Paired comparison of IC50 values for insulin and proinsulin.** IC50 values (nM) were calculated for the displacement of IAA binding by insulin and proinsulin in the ADAP assay. Each line represents an individual subject. Boxplots summarize the distribution of IC50 values; the *y*-axis is displayed on a log10 scale. Median IC50 values were 21.0 nM (IQR 14.9–25.1) for insulin and 26.1 nM (IQR 12.3–37.5) for proinsulin. In paired analyses on log10-transformed IC50 values, the difference between ligands did not reach statistical significance (Wilcoxon signed-rank test, *P* = 0.081).

Displacement curves demonstrated concentration-dependent inhibition for both ligands (Fig. 1a–b, Tables 2–3). Proinsulin curves were consistently right-shifted relative to insulin, indicating that higher concentrations of proinsulin were required to achieve comparable displacement, consistent with lower binding potency. This visual trend was reflected in IC50 analysis: median IC50 was 21.0 nM (IQR 14.9–25.1) for insulin and 26.1 nM (IQR 12.3–37.5) for proinsulin (Fig. 2). Proinsulin IC50 values showed greater variability (median fold difference 1.23). Median log10 IC50 values were 1.32 (IQR 1.17–1.40) for insulin and 1.42 (IQR 1.09–1.57) for proinsulin.

**Table 3:** ADAP IAA (Δct) with and without displacement with 0–53 nM recombinant human proinsulin.

Sample number	Proinsulin displacement concentration (nM)				
	0	0.053	0.53	5.3	53
01	4.44	4.03	3.83	2.69	0.78
02	5.39	4.26	4.52	2.48	1.59
03	7.17	5.77	5.48	4.43	2.15
04	7.63	6.19	5.93	3.71	2.20
05	6.94	6.30	5.23	5.12	3.14
06	7.47	5.68	5.91	5.12	3.18
07	4.76	4.34	3.95	3.39	2.79
08	5.10	4.89	4.89	4.64	5.06
09	3.60	3.25	2.59	1.89	1.46
10	5.68	4.46	3.05	1.50	0.29
11	6.97	6.59	6.11	4.45	3.84
12	4.98	4.73	4.01	2.56	2.70
13	5.47	5.07	4.27	4.06	2.83
14	5.49	4.93	3.45	2.69	2.54
15	6.19	5.78	4.92	3.70	2.33
16	7.56	6.59	6.27	5.46	5.39
17	3.22	3.40	2.73	1.01	−0.24
18	4.91	4.26	4.40	3.28	2.18
19	5.79	5.63	4.62	3.75	1.68
20	4.03	3.59	3.19	2.46	2.41
21	4.06	3.82	4.68	3.29	1.90
22	6.24	6.03	6.00	4.68	3.33
23	4.82	4.62	4.44	3.60	3.55
24	7.70	6.37	6.12	5.46	4.36
25	6.45	6.32	4.91	3.55	2.33
26	6.42	5.62	5.47	4.09	3.64
27	7.84	7.52	5.95	4.33	1.85
28	7.45	6.60	6.18	4.53	3.52
29	6.55	6.02	4.70	4.03	3.18
30	4.68	3.73	4.21	3.39	2.67
31	4.35	4.07	3.57	1.91	1.45
32	7.36	6.40	5.84	4.09	2.72
33	6.57	4.99	5.08	3.83	3.81
34	8.78	7.22	8.07	5.71	4.82
35	7.92	8.71	8.17	6.42	4.97
36	6.97	5.53	5.58	3.96	3.78

In paired analyses on the log10 scale, proinsulin IC50 values were slightly higher than insulin IC50 values, though the difference did not reach statistical significance (Wilcoxon signed-rank test, *P* = 0.081; Table 4, Fig. 2). Median within-individual Δlog10(IC50) was 0.088 (IQR −0.050 to 0.237), indicating broadly similar binding characteristics between insulin and proinsulin across participants.

**Table 4:** Comparison of insulin and proinsulin IC50 values and their within-individual differences by HLA-DQ genotype and autoantibody status.

Sample number	Sex	HLA-DQ genotype	AAb status	Log10 iC50 insulin	Log10 iC50 proinsulin	Δlog10 iC50 (PINS-INS)	IC50 insulin	IC50 proinsulin	Fold difference (PINS/INS)
01	F	DQ2/DQ8	4 AAbs	1.189	1.841	0.652	15.442	69.348	4.491
02	M	DQ2/DQ8	3 AAbs	1.395	1.451	0.056	24.825	28.221	1.137
03	F	DQ8 only	3 AAbs	1.444	1.609	0.165	27.812	40.643	1.461
04	F	DQ2/DQ8	3 AAbs	1.141	1.416	0.275	13.831	26.074	1.885
05	M	DQ8 only	4 AAbs	1.06	1.837	0.777	11.483	68.682	5.981
06	F	DQ2/DQ8	4 AAbs	1.395	1.589	0.194	24.838	38.823	1.563
07	F	DQ8 only	3 AAbs	1.214	1.291	0.077	16.369	19.562	1.195
09	M	DQ2/DQ8	4 AAbs	0.738	0.971	0.234	5.465	9.357	1.712
10	M	DQ8 only	3 AAbs	0.408	0.868	0.461	2.558	7.387	2.888
11	F	DQ2 only	4 AAbs	1.303	1.391	0.088	20.088	24.611	1.225
12	F	DQ8 only	2 AAbs	0.881	1.112	0.231	7.609	12.948	1.702
13	F	DQ2 only	2 AAbs	1.42	1.494	0.074	26.324	31.214	1.186
14	F	DQ8 only	3 AAbs	1.323	0.578	−0.745	21.049	3.782	0.18
15	F	DQ2 only	4 AAbs	0.804	1.475	0.671	6.361	29.844	4.692
16	M	DQ8 only	2 AAbs	1.482	0.418	−1.064	30.311	2.617	0.086
17	M	DQ2 only	3 AAbs	1.316	1.551	0.236	20.682	35.587	1.721
18	M	DQ2/DQ8	4 AAbs	1.289	1.6	0.312	19.45	39.853	2.049
19	F	DQ8 only	3 AAbs	1.387	1.73	0.344	24.367	53.761	2.206
20	M	DQ8 only	4 AAbs	1.169	0.713	−0.456	14.745	5.162	0.35
21	F	DQ8 only	3 AAbs	1.375	1.703	0.328	23.733	50.5	2.128
22	M	DQ2/DQ8	4 AAbs	1.459	1.671	0.212	28.788	46.92	1.63
23	M	DQ2 only	3 AAbs	1.4	1.282	−0.117	25.111	19.161	0.763
24	F	DQ2/DQ8	3 AAbs	1.401	1.066	−0.335	25.15	11.636	0.463
25	M	DQ2/DQ8	4 AAbs	1.251	1.356	0.105	17.825	22.706	1.274
26	F	DQ8 only	4 AAbs	1.364	1.298	−0.067	23.132	19.848	0.858
27	M	DQ2 only	3 AAbs	1.47	1.559	0.088	29.534	36.194	1.226
28	M	DQ2/DQ8	4 AAbs	1.387	1.388	0.002	24.351	24.455	1.004
29	M	DQ2/DQ8	4 AAbs	0.979	0.694	−0.286	9.536	4.938	0.518
30	F	Neither	3 AAbs	1.463	1.542	0.079	29.055	34.86	1.2
31	M	DQ2/DQ8	3 AAbs	1.282	1.374	0.092	19.133	23.663	1.237
32	F	DQ2/DQ8	4 AAbs	1.178	1.416	0.238	15.059	26.071	1.731
33	M	DQ2/DQ8	4 AAbs	1.34	−0.028	−1.368	21.879	0.937	0.043
34	F	Unknown	2 AAbs	1.551	1.473	−0.078	35.582	29.716	0.835
35	F	DQ8 only	≥3 AAbs*	1.597	1.599	0.002	39.56	39.7	1.004
36	F	DQ2/DQ8	≥3 AAbs*	1.082	1.049	−0.033	12.084	11.194	0.926

IC50 values are log10-transformed. Δlog10 IC50 = log10(PINS IC50) − log10(INS IC50). Fold difference = PINS IC50/INS IC50. F, female; M, male.

### Age- and sex-related associations

**Figure 3:**
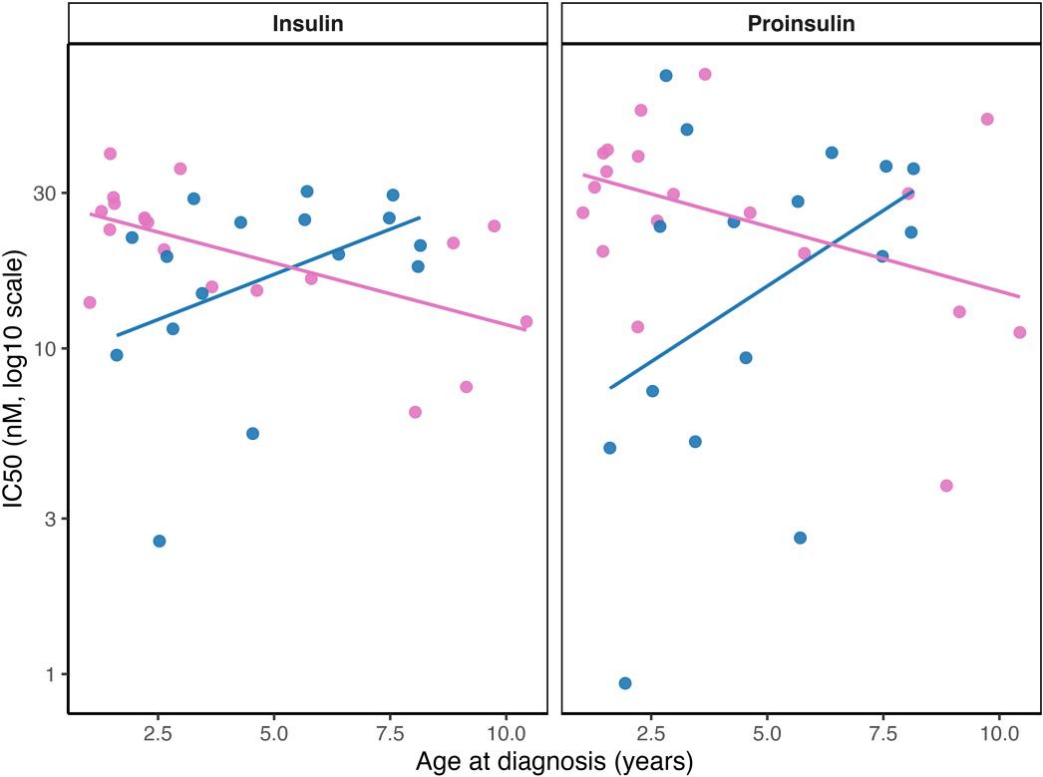
**Associations between age at diagnosis and IC50 values for insulin and proinsulin.** Scatterplots show log10-transformed IC50 values plotted against age at diagnosis, separately for insulin and proinsulin. Blue dots indicate boys; pink dots indicate girls. Linear mixed-effects models including a random intercept for sample number were used to account for repeated measurements. No overall association between IC50 and age was observed. However, a significant age × sex interaction was detected (F (1,31) = 14.3, *P* < 0.001), indicating that IC50 increased with age in boys and decreased with age in girls. No interaction between age and ligand type was observed. Trendlines are shown separately for boys and girls for visualization purposes.

Linear mixed-effects models accounting for repeated measures revealed no overall association between IC50 and age at diagnosis (*F* (1,31) = 1.39, *P* = 0.247). However, a significant age × sex interaction was observed (*F* (1,31) = 14.3, *P* < 0.001), indicating that IC50 increased with age in boys but decreased in girls. Patterns were consistent for both ligands (all ligand × age interactions *P* > 0.27; Fig. 3).

**Figure 4:**
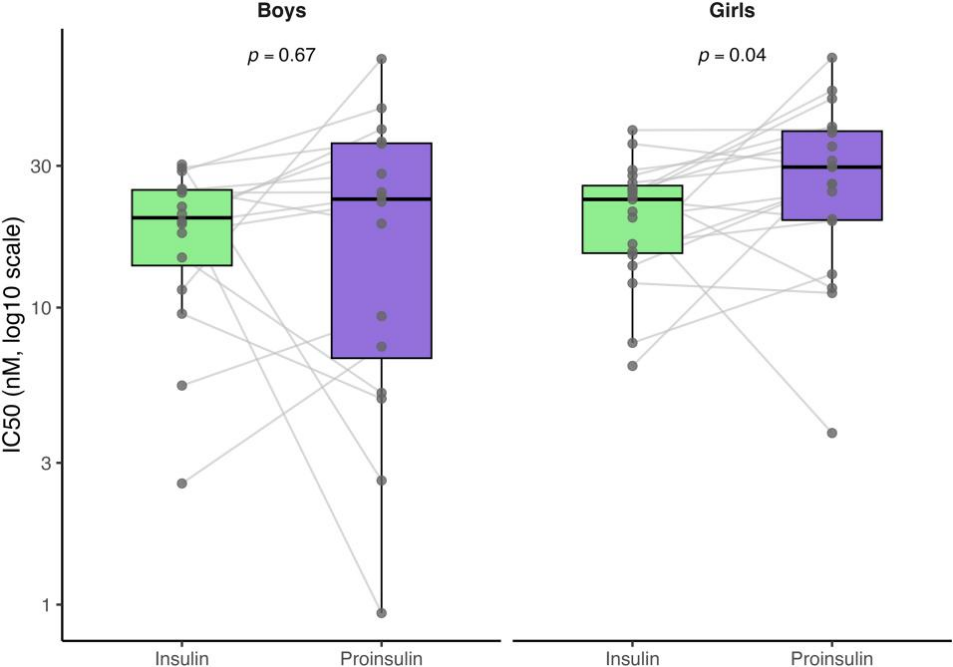
**IC50 values for insulin and proinsulin stratified by sex.** Paired IC50 values (nM; *y*-axis displayed on a log10 scale) for insulin and proinsulin are shown separately for boys and girls. Individual subjects are connected by grey lines, and boxplots summarize the distribution within each sex. Median IC50 values in girls were 23.1 nM (IQR 15.3–25.7) for insulin and 29.7 nM (19.7–39.3) for proinsulin. In boys, corresponding values were 20.1 nM (13.9–24.9) and 23.2 nM (6.83–35.7), respectively. Proinsulin IC50 values showed greater variability among boys compared with girls. Within-individual ligand differences were assessed using Wilcoxon signed-rank tests on log10-transformed IC50 values. A significant difference between insulin and proinsulin IC50 was observed in girls (*P* = 0.04, n = 19) but not in boys (*P* = 0.67, n = 16).

Within-individual comparisons showed IC50 differences between insulin and proinsulin in girls (Wilcoxon signed-rank test, *P* = 0.04, n = 19) but not in boys (*P* = 0.67, n = 16). Median IC50 in girls was 23.1 nM (15.3–25.7) for insulin and 29.7 nM (19.7–39.3) for proinsulin; in boys, 20.1 nM (13.9–24.9) and 23.2 nM (6.83–35.7), respectively (Fig. 4).

### Associations with HLA and autoantibody status

**Figure 5:**
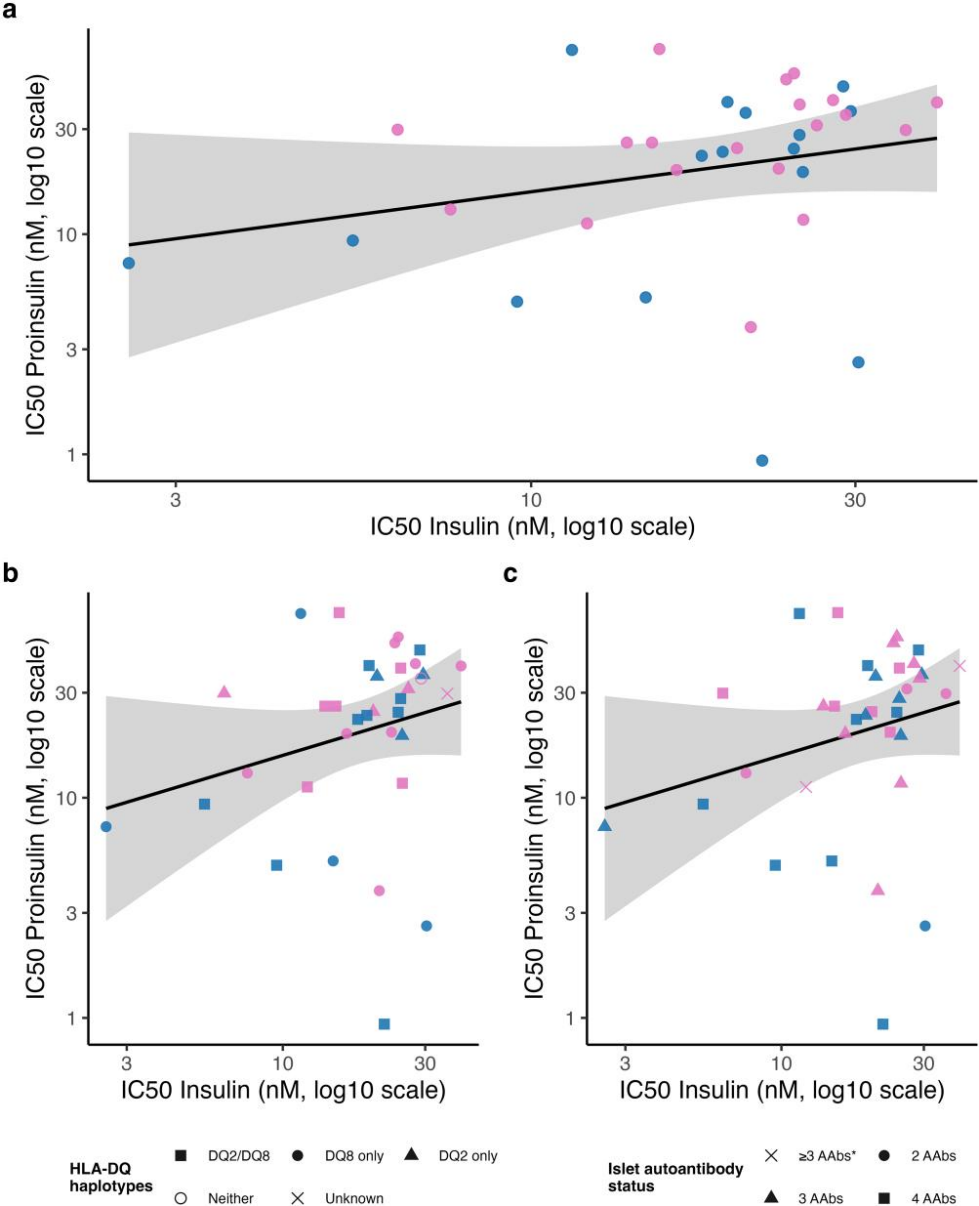
**IC50 values in relation to sex, HLA genotype, and autoantibody status.** All IC50 values are shown on a log10 scale. Blue dots indicate boys; pink dots indicate girls. (a) Scatterplot of paired log10(IC50) values for insulin and proinsulin, colored by sex. In linear regression, log10(IC50_proinsulin) was not significantly associated with log10(IC50_insulin) (β = 0.14, SE = 0.10, *P* = 0.16), and adjustment for sex did not materially change the estimate (*P* = 0.24). (b) IC50 values stratified by HLA-DQ genotype. Points are shaped according to HLA group (DQ2/DQ8 (n = 15), DQ2 only (n = 6), DQ8 only (n = 12), neither (n = 1), unknown (n = 1)). No significant association was observed between HLA genotype and IC50 (overall model *P* = 0.60). **(**c**)** IC50 values stratified by number of islet autoantibodies (AAb). Points are shaped according to AAb status (4 (n = 15), 3 (n = 14), 2 (n = 4), or ≥3 AAbs* (n = 2)). No significant association was observed between autoantibody status and IC50 (overall model *P* = 0.44). Sensitivity analyses excluding individuals with incomplete autoantibody data or using alternative groupings yielded similar results. ≥ 3 AAbs*: This group includes two subjects with missing ZnT8A data. Both had three confirmed autoantibodies; however, a fourth autoantibody could not be excluded.

IC50 values for insulin and proinsulin were modestly associated but not statistically significant (β = 0.14, SE = 0.10, *P* = 0.16; Table 1, Fig. 5a). This relationship was not modified by sex, HLA genotype, or autoantibody status (all *P* > 0.24; Fig. 5b-c). Sensitivity analyses excluding participants with ambiguous autoantibody data or collapsing autoantibody groups produced similar results, supporting robustness.

Within-individual Δlog10(IC50) values were also examined. These were not associated with sex (*P* = 0.61), HLA genotype (*P* = 0.89), or autoantibody status (*P* = 0.39; Table 4), indicating consistent binding characteristics across subgroups.

### Relationship between ADAP IAA levels and IC50

**Figure 6:**
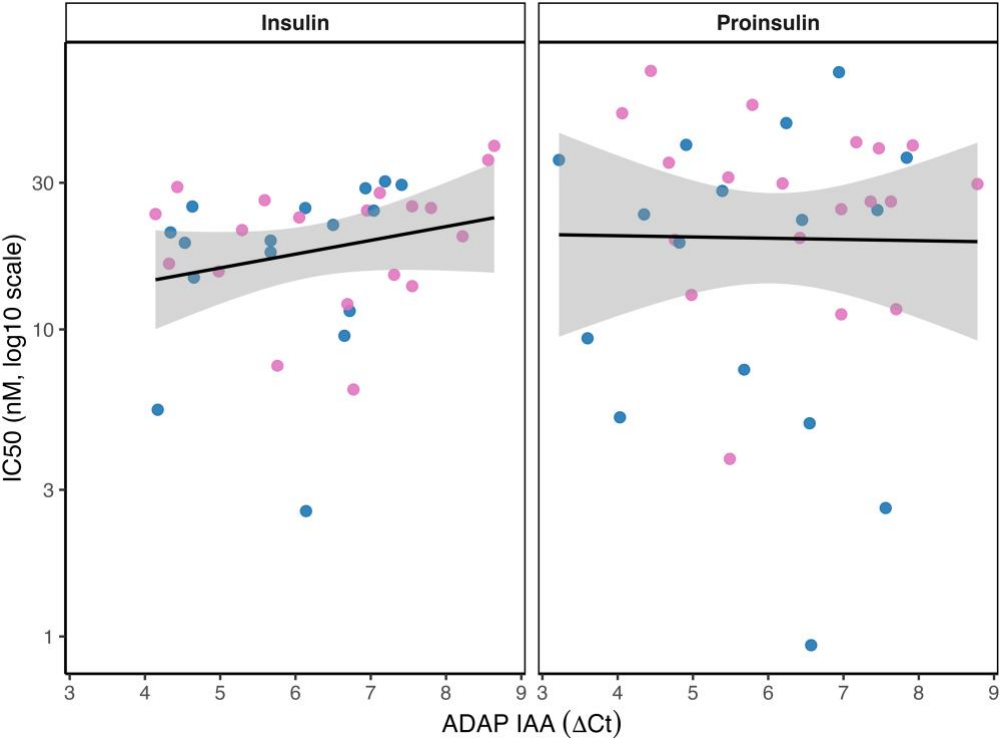
**Association between ADAP IAA levels and IC50 for insulin and proinsulin.** Scatterplots show the relationship between ADAP IAA levels (ΔCt) and log10-transformed IC50 values (nM) for insulin (left) and proinsulin (right). Each point represents one participant. Blue dots indicate boys; pink dots indicate girls. Mixed-effects linear regression models including a random intercept for sample number were used to account for repeated measurements. No significant association between ADAP IAA levels and IC50 was observed (β = 0.039, SE = 0.045, *P* = 0.39).

In mixed-effects regression analyses accounting for repeated measures, ADAP IAA levels were not associated with log10(IC50) (β = 0.039, SE = 0.045, *P* = 0.39). There was no evidence of a differential association by ligand (interaction β = −0.046, SE = 0.056, *P* = 0.41; Fig. 6).

## Discussion

In this study of 36 newly diagnosed T1D children randomly selected from a prior investigation of ADAP-IAA [19], it was found that the average affinity was higher for insulin compared to proinsulin. All samples except possibly one sample were displaced by both insulin and proinsulin. Overall, this may be taken as an indication that insulin is the primary autoantigen. When comparing the individual IC50 values for insulin and proinsulin, a considerable heterogeneity in the competition between insulin and proinsulin for IAA was discovered. While insulin and proinsulin were comparable in most samples, there were 10 children with an increased ability of proinsulin over insulin to displace IAA. Taken together, these data indicate that IAA has a greater affinity for insulin compared to proinsulin, except for some patients similar to a report using the ^125^I-insulin assay [11]. The heterogeneity between insulin and proinsulin displacement in the paired samples does not allow a conclusion that insulin is the primary target autoantigen.

The strength of this study is that all children had only been treated with insulin for 3–5 days, indicating that insulin antibodies were not yet formed to interfere with the data. The IC50-INS values suggested higher affinity for IAA in the young age-at-onset children, which would favor insulin as the primary antigen as the time between the presumed first appearance of IAA and clinical onset is shorter. In the TEDDY study, IAA-first was more common among boys [3]. IAA as the first appearing autoantibody is associated with HLA DR4-DQ8 [3, 11] but there was no indication that this association was detected at the time of clinical onset of type 1 diabetes. Neither did HLA DQ-haplotype and the presence of other islet autoantibodies correlate with IC50, which supports the view that the appearance of a second or third islet autoantibody was not associated with HLA [6]. The significant correlation between ADAP-IAA and IC50-insulin, but not IC50-proinsulin further supports the idea that insulin may be the primary autoantigen, as proinsulin is a poorer competitor with lower potency.

Sample number 08 was the only sample that was not displaced by proinsulin. The sample was from a girl who was 7.89 years old at diagnosis (Table 1). Her ADAP IAA levels were 8.01 ΔCt, and she had the HLA DQ2/DQ8 genotype (Table 1). Besides IAA, she was also positive for GADA but negative for IA-2A and ZnT8A (Table 1). These characteristics are not sufficiently different from the other children to draw a conclusion about why this child would be insulin-specific without her IAA cross-reacting with proinsulin.

IAA in children usually develops long before they are diagnosed with autoimmune diabetes [3, 23]. There is a lack of information on how insulin is presented to the immune system to initiate IAA. Studies of T lymphocytes in children at risk or with type 1 diabetes have shown that there are T-cell receptors that recognize proinsulin epitopes rather than insulin peptide epitopes [24].

A weakness of the present investigation was that oligonucleotide-conjugated proinsulin was not available as the labeled antigen, which would have made a reciprocal analysis possible. For example, the heterogeneity in the study group may be a result of epitope spreading during the often-slow progression towards the clinical onset. It is assumed that the immune system destroys beta cells, leading to a booster effect of insulin as well as proinsulin as the autoantigen. The initial immune response to insulin may mature through an increasing affinity of proinsulin for IAA.

Some previous studies had shown that proinsulin was the main targeted autoantigen [9], while others reported that insulin was more specific [10]. The main discovery of our study using the best available assay for IAA [15] was that most of the samples from the newly diagnosed type 1 diabetes patients were displaced better by insulin than proinsulin. In addition, a substantial heterogeneity in the competition between insulin and proinsulin to bind IAA was discovered in these patients. The findings of this study open possibilities for future research with the aim of finding out in children followed from birth [2, 3, 25] if it is possible to distinguish insulin from proinsulin as the primary target autoantigen.

Further research needs to determine the role of proinsulin as an autoantigen in different stages of type 1 diabetes. The patients of this study were at the latest stage of their pathogenesis, recently referred to as stage 3 type 1 diabetes [26]. Therefore, the results cannot be applied to patients at all stages of type 1 diabetes. It would be of great interest to test the displacement of insulin and proinsulin in individuals at earlier stages of the disease, similar to a previous study with IAA detected in the ^125^I-insulin radioimmunoassay [11, 12]. A critical analysis would be to analyze the very first appearing IAA in the TEDDY study [3] using both insulin and proinsulin conjugated with oligonucleotides in a custom-designed multiplex ADAP [16, 19].

## Conclusion

In summary, it is concluded that insulin is likely to be the primary autoantigen when compared to proinsulin in most of the patients with newly diagnosed type 1 diabetes. Understanding of these components of the autoimmune response is critical to improve IAA as an early biomarker of the initiation of the pathogenesis to develop immunotherapies that could delay the onset or even cure the disease.

## Data Availability

All data will be made available from J.J., P.P., or Å.L. after a reasonable request.
